# Influence of voxel size on the accuracy of linear measurements of the condyle in images of cone beam computed tomography: A pilot study

**DOI:** 10.4317/jced.54500

**Published:** 2018-09-01

**Authors:** Andre-Luiz-Ferreira Costa, Bruna-Viana Barbosa, João-Pedro Perez-Gomes, Alison-Jhisel-Mansmith Calle, Mauro-Pedrine Santamaria, Sérgio-Lúcio-Pereira-de Castro Lopes

**Affiliations:** 1 Department of Orthodontics and Radiology, University of São Paulo City (UNICID), São Paulo, SP, Brazil; 2 Department of Diagnosis and Surgery, São José dos Campos Dental School, São Paulo State University (UNESP), São José dos Campos, SP, Brazil; 3 Department of Internal Medicine, Faculty of Medical Sciences, University of Campinas (UNICAMP), Campinas, São Paulo, Brazil

## Abstract

**Background:**

To analyze the influence of voxel size and exposure time on the accuracy of linear measurements of the condyle.

**Material and Methods:**

Four macerated hemi-mandibles of pigs were scanned in nine different voxel size protocols. Three-dimensional models of the condyle were generated in order to establish a comparison between linear measurements obtained with each voxel protocol and those obtained with a caliper (gold standard). The comparison between the protocols was performed considering the average of the two measurements of the condyle in the latero-medial (LM) and antero-posterior (AP) axes and also through repeated measurement ANOVA with rank transformation. The level of significance was 5%.

**Results:**

A significant difference was found between the protocols regarding the LM and AP variables (p-values = 0.0027 and 0.0263, respectively). In the LM axis, the protocol P6 (voxel size of 0.3 mm with scan time of 4.8 seconds) did not show statistical difference compared to the gold standard. The protocols P4 and P5 (voxel size of 0.25 mm with scan times of 14.7 and 26.9 seconds, respectively) were both statistically similar compared to caliper, although they have presented a longer scan time. In the AP axis, the protocol P8 (voxel size of 0.4 mm with time scan of 4.8 seconds) was statistically similar to the gold standard.

**Conclusions:**

A smaller voxel size does not necessarily mean more accuracy regarding the linear measurements of the condyle. It is possible to obtain an acceptable level of accuracy with a larger voxel size and a shorter exposure time to radiation.

** Key words:**CBCT, voxel size, linear measurement, diagnostic imaging.

## Introduction

Cone beam computed tomography (CBCT) has been reported as an imaging technique capable of providing benefits in a wide range of areas. It is possible to exemplify the recommendation of CBCT in a variety of situations, since to properly detect the genial spinal canal during pre-surgical diagnostics (1), to help in the evaluation and treatment planning in dental implantology. CBCT has proven to be of great importance as well in temporomandibular joint (TMJ) assessment and in oral and maxillofacial surgery (2).

The voxel size in CBCT is smaller than in conventional computed tomography and might variate depending upon the chosen protocol (3). A smaller voxel size may be associated with a longer scan time, which may be related to some undesirable situations, such as increased possibility of patient movement during the procedure, higher radiation doses and longer reconstruction time (3).

Reducing the voxel size results in increased spatial resolution. However, the use of a smaller voxel size results in a higher dose of radiation (4). Considering the voxel size is related to the ionizing radiation dose supplied to the patient, it certainly deserves special consideration.

It has been hypothesized that CBCT image voxel size is inversely related to the ability to detect osseous changes observed in degenerative joint disease of TMJ (5). However, no significant differences were found in the abilities of oral and maxillofacial radiologists to detect osseous changes using different voxel size protocols (5).

To the best of our knowledge, there is no similar study addressing the relation between the linear measurements of the mandibular condyle with the voxel size variation and, consequently, the exposure time to ionizing radiation. Therefore, the aim of this work consisted in analyzing the influence of voxel size for accurately stablish the linear measurements between the antero-posterior (AP) and latero-medial (LM) points of the condyle. By doing so, we stablished the protocols able to provide an acceptable reliability of the measures in question, but at the same time, reducing the collateral damage, preventing the patient from receiving unnecessary radiation dose.

## Material and Methods

Data collection for this study was approved by the Institute of Science and Technology of the Paulista State University Institutional Review Board. The sample consisted in 04 intact swine macerated hemi-mandibles. No history of bone disease was previously detected in the animals. Swine mandibles have been used in several studies, including the comparison between CBCT and conventional intraoral radiographs in detecting interproximal alveolar bone lesions (6). Also, swine heads have been used to compare CBCT with multislice computed tomography in detection of small osseous condylar defects. The swine condyles were useful to conclude that orthodontic-grade CBCT images of mandibular condyle may be less reliable and less accurate for the diagnosis of small condylar defects, even at a lower voxel size (7).

**Figure 1:**
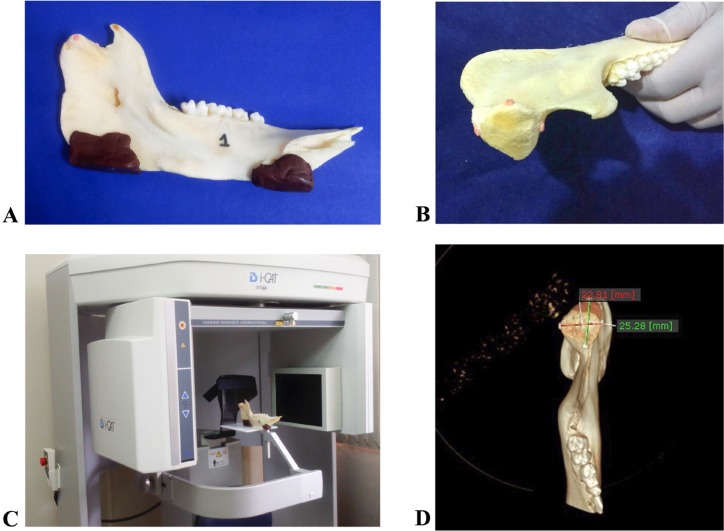
A) Identified macerated porcine hemi-mandible with thermo-heated godiva base for stabilization on the I-CAT Next Generation brand scanner. B) Marking points using heated gutta-percha measuring 0.2 mm of diameter in the macerated porcine hemi-mandible´s condyle. Each gutta-percha point represents the most prominent area of the condyle´s medial and lateral poles and anterior and posterior strands. C) Sample positioned and stabilized on a flat support on the I-CAT Next Generation scanner. D) Three-dimensional model generated in multiplanar reconstruction windows (MPR), in which bone tissue protocols were applied. All images were exported in Digital Imaging and Communications in Medicine format to OnDemand3D software (Cybermed, Tustin, CA, USA).

The samples were numbered from 01 (Fig. 1) to 04. In each sample was demarcated 04 points using heated gutta-percha measuring 0.2mm of diameter in the condyle (Fig. 1). The gutta-percha has already been considered necessary for CBCT images to indicate the sites of interest (8).

The demarcated points corresponded to:

M – Most prominent area of condyle medial pole;

L – Most prominent area of condyle lateral pole;

A – Most prominent area of condyle anterior strand.

P – Most prominent area of condyle posterior strand;

The samples were individually positioned on the I-CAT Next Generation scanner (Imaging Sciences International, Hatefiled, PA, USA) (Fig. 1). On a flat support and stabilized by a thermo-heated godiva base (Figure 1), the acquisitions of each hemi-mandible were performed according to the protocol shown in  Table 1.

**Table 1:**
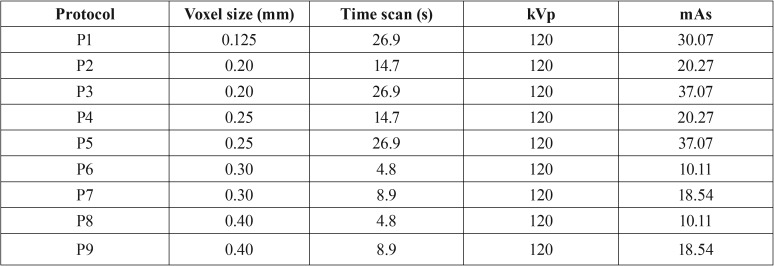
Distribution of protocols according to voxel size, time scan, kilovolt peak and milliamps x seconds. mm – millimeters; s – seconds.

The Field of View (FOV) used was 16×6 cm. All images were exported in DICOM (Digital Imaging and Communications in Medicine) format to OnDemand3D software (Cybermed, Tustin, CA, USA). Three-dimensional (3D) models were generated in the multiplanar reconstruction in which bone tissue protocols were applied.

Under dim lighting conditions, images were assessed by one previously calibrated oral radiologist following the protocol:

A) With the 3D ruler tool of the software, which allowed to perform linear measurements on the 3D images, the following linear measurements between “L” and “M”, as well as “A” and “P”, were obtained from each image in each protocol (Fig. 1).

B) Subsequently, in each mandible individually was measured the linear distance between points LM and AP, using a caliper.

All the values were tabulated and submitted to statistical analysis in order to verify whether there was a significant difference between the accuracy of different groups of voxel size protocols and the data provided by the caliper (Vernier Caliper – Standard Model; Graduation: 0.05 mm, 0.05 mm; Mitutoyo, U.S.A.), which corresponded to the gold standard.

After 21 days, the sample was re-evaluated in the same manner for assessment of the reproducibility of the method.

The exploratory analysis of the data was performed using summary measures (average, standard deviation, minimum, median and maximum) and constructed graphics. The comparison between the protocols was performed considering the average of the two measures and through ANOVA for repeated measures with rank transformation. The level of significance was 5%.

## Results

The descriptive statistics of difference between time of the measurement in LM and AP variables are detailed in  Table 2.

**Table 2:**
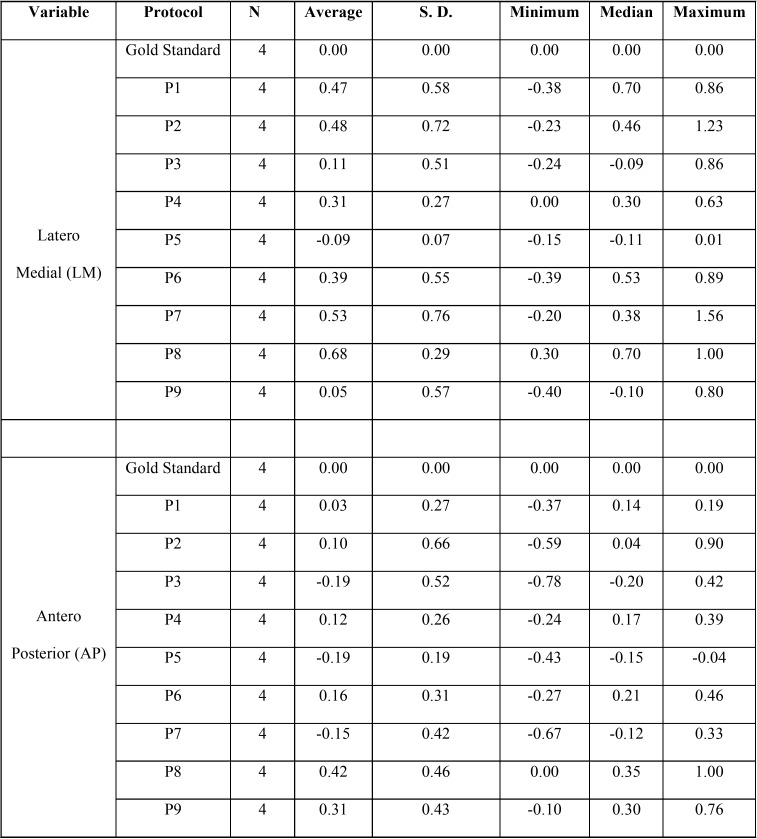
Descriptive statistics of difference between time of the measurement in LM and AP variables. N – Sample size; S.D. – Standard deviation.

Summaries measures between the two repetitions for LM and AP variables with result of the comparison between each protocol is detailed in  Table 3 and  Table 4respectively. There was found significant difference between the protocols in LM and AP variables (*p*-value = 0.0027) and (*p*-value = 0.0263) respectively.

**Table 3:**
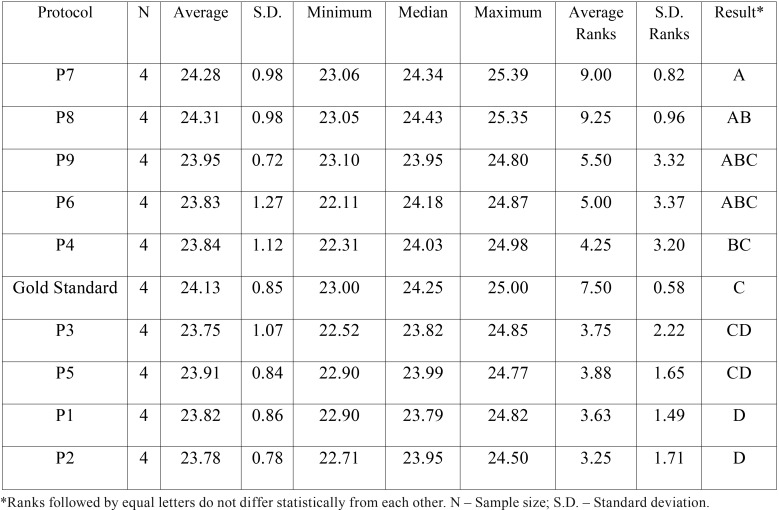
Summaries measures between the two repetitions in Latero-Medial variable and result of the comparison between each protocol by rank transformation.

**Table 4:**
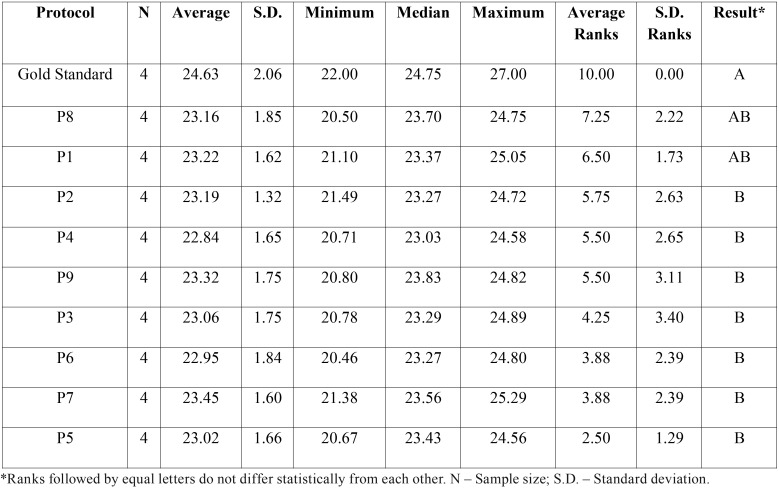
Summaries measures between the two repetitions in Antero-Posterior variable and result of the comparison between each protocol by rank transformation.

In LM, the protocol P6 (voxel size 0.3 mm with 4.8 seconds of time scan) presented similar measures compared to the gold standard. The same happened with the protocols P3, P4, P5 and P9. However, these protocols present a smaller voxel size, or even, a longer exposure time to ionizing radiation. On the other hand, in AP variable, the protocol P8 (voxel size 0.4 mm with 4.8 seconds of time scan) presented better results over the others, even considering the protocol P9, which is a protocol with the same voxel size as P8, but with a longer exposure time.

**Figure 2:**
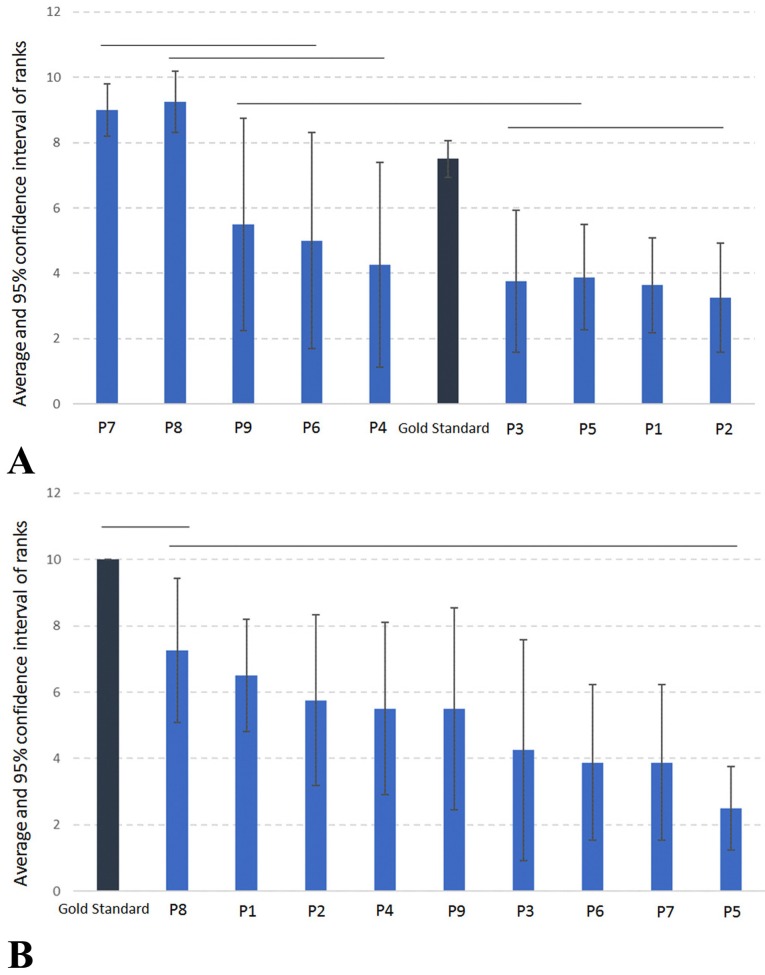
A) Average and 95% confidence interval of ranks in each protocol considering Latero-Medial measure. P1 – voxel size 0.125 mm with time scan 26.9 seconds; P2 – voxel size 0.20 mm with time scan 14.7 seconds; P3 – voxel size 0.20 mm with time scan 26.9 seconds; P4 – voxel size 0.25 mm with time scan 14.7 seconds; P5 – voxel size 0.25 mm with time scan 26.9 seconds; P6 – voxel size 0.30 mm with time scan 4.8 seconds; P7 – voxel size 0.30 mm with time scan 8.9 seconds; P8 – voxel size 0.40 mm with time scan 4.8 seconds; P9 – voxel size 0.40 mm with time scan 8.9 seconds. B) Average and 95% confidence interval of ranks in each protocol considering Antero-Posterior measure. P1 – voxel size 0.125 mm with time scan 26.9 seconds; P2 – voxel size 0.20 mm with time scan 14.7 seconds; P3 – voxel size 0.20 mm with time scan 26.9 seconds; P4 – voxel size 0.25 mm with time scan 14.7 seconds; P5 – voxel size 0.25 mm with time scan 26.9 seconds; P6 – voxel size 0.30 mm with time scan 4.8 seconds; P7 – voxel size 0.30 mm with time scan 8.9 seconds; P8 – voxel size 0.40 mm with time scan 4.8 seconds; P9 – voxel size 0.40 mm with time scan 8.9 seconds.

The average and 95% confidence interval of ranks in each protocol considering LM and AP measures are detailed respectively in Figure 2.

Our results emphasize that a smaller voxel size and an increased exposure time do not necessarily mean a greater accuracy with regard to the linear measurements analysis of the mandibular condyle. It is possible to obtain a reliable diagnosis using a larger voxel size and a shorter exposure time to radiation.

## Discussion

3D-CBCT images have been suggested as a way to obtain dimensionally accurate linear and angular measurements from bony maxillofacial structures (9).

However, there is not as much consensus regarding the voxel protocol to be recommended for certain situations. For example, in implantology expertise, it has been shown that lowering the CBCT exposure time in imaging of dry skulls does not affect the accuracy of implant site measurements (10).

The aim of this study was to evaluate the capability of different voxel size protocols in accurately obtaining the linear measurements of the condyle poles. By doing so, we were able to determine, basing on the present sample, that it is possible to achieve a reliable diagnosis by using a lesser amount of radiation in the CBCT-scan process.

Our results show that it is possible to obtain accuracy regarding the linear measurements of the condyle by using a larger voxel size protocol and, therefore, prevent the patient from receiving an unnecessary radiation dose.

Considering the LM variable, the protocol P6 deserves especial consideration. It has presented the same accuracy as the caliper, but presented also a larger voxel size than the protocols P3, P4 and P5. At the same time, presented a shorter exposure time than P9 and a greater reliability when compared to P1, P2, P7 and P8.

Considering the AP variable, the protocol to be highlighted is P8. It was statistically similar to the gold standard and presented with greater accuracy, even when compared to P9, which is a protocol of same voxel size, but with a longer exposure time.

Potential limitation of the study includes small sample size. Moreover, since only one radiologist participated in the assessment, different results might have been seen if more examiners had been involved. However, despite these limitations, the present study provides valuable information.

This is a pilot study in which we present indications that it is possible to achieve an acceptable level of accuracy of the condyle´s linear measurements by using a larger voxel size CBCT protocol and by consequence, reducing the amount of ionizing radiation supplied to the patient.
